# Chromosome-Level Genome Assembly of *Apoderus dimidiatus* Voss (Coleoptera: Attelabidae): Insights into Evolution and Behavior

**DOI:** 10.3390/insects15060431

**Published:** 2024-06-06

**Authors:** Meng Xie, Yuhao Yao, Yuling Feng, Lei Xie, Chuyang Mao, Jinwu He, Xueyan Li, Qingyong Ni

**Affiliations:** 1 College of Life Science, Sichuan Agricultural University, Ya’an 625014, China; xiemeng@sicau.edu.cn (M.X.); yaoyh18981108390@163.com (Y.Y.); 2 Key Laboratory of Livestock and Poultry Multi-Omics, Ministry of Agriculture and Rural Affairs, College of Animal Science and Technology, Sichuan Agricultural University, Chengdu 611130, China; fengyuling0@outlook.com (Y.F.); xl2449616395@outlook.com (L.X.); 3 State Key Laboratory of Genetic Resources and Evolution, Kunming Institute of Zoology, Chinese Academy of Sciences (CAS), Kunming 650223, China; maochuyang@mail.kiz.ac.cn (C.M.); hejinwu@mail.kiz.ac.cn (J.H.)

**Keywords:** genome, weevil species, expansion and contraction, positive selection

## Abstract

**Simple Summary:**

*Apoderus dimidiatus* (Coleoptera: Attelabidae), a widely distributed weevil species in China, feeds on leaves and creates compact leaf rolls for spawning. Its offspring complete a series of developmental stages within these structures before emerging as mature insects. In this study, we assembled a high-quality genome of *A. dimidiatus* at the chromosome level. We found the expanded gene family of *A. dimidiatus* is associated with functions such as the regulation of skin water loss, sebaceous gland development, and water balance in multicellular organisms, which are essential for the species’ survival within leaf rolls. On the other hand, the contracted gene family is primarily associated with protease activity and solute transmembrane transport, contributing to the decline in egg production and the low survival rate of offspring. This situation forces female adults to develop the unique behavior of leaf rolling. Overall, our findings shed light on the evolutionary relationship between *A. dimidiatus* and other species and elucidate the molecular mechanisms behind their unique leaf rolling behavior.

**Abstract:**

Attelabidae insects have attracted much attention due to their unique leaf rolling behavior before oviposition. However, the lack of genomic data makes it difficult to understand the molecular mechanism behind their behavior and their evolutionary relationship with other species. To address this gap, we utilized Illumina and Nanopore sequencing platforms along with Hi-C technology to establish a highly accurate whole genome of *A. dimidiatus* at the chromosome level. The resulting genome size was determined to be 619.26 Mb, with a contig N50 of 50.89 Mb and GC content of 33.89%. Moreover, a total of 12,572 genes were identified, with 82.59% being functionally annotated, and 64.78% designated as repeat sequences. Our subsequent phylogenetic tree analysis revealed that Attelabidae’s divergence from Curculionidae occurred approximately 161.52 million years ago. Furthermore, the genome of *A. dimidiatus* contained 334 expanded gene families and 1718 contracted gene families. In addition, using Phylogenetic Analysis by Maximum Likelihood (PAML), we identified 106 rapidly evolved genes exhibiting significant signals and 540 positively selected genes. Our research endeavors to serve as an invaluable genomic data resource for the study of Attelabidae, offering fresh perspectives for the exploration of its leaf rolling behavior.

## 1. Introduction

The continuous improvement of sequencing technology and related analytical methods has propelled life science into the genomics era, thereby bringing new perspectives to the field of entomology. Researchers have expanded their understanding of insects beyond behavioral and morphological levels by incorporating molecular genetic analysis. The sequencing of several insect genomes has been pivotal in advancing this endeavor: *Drosophila melanogaster* Meigen [1], *Anopheles gambiae* Giles [2], *Bombyx mori* Linnaeus [3], *Apis mellifera* Linnaeus [4], *Aedes aegypti* Liston [5], *Tribolium castaneum* Herbst [6], *Apoderus coryli* Lmnaeus [7], etc. The analysis of these genomes has laid the groundwork for a new era in insect science, providing a valuable resource for the development of alternative and environmentally friendly pest control strategies. Comparative genomics has contributed essential data for studying genome structure variation and genetic mechanisms. It has been instrumental in unraveling the diverse origins of critical insect behaviors, serving as a potent method for identifying new targets for pest control.

Attelabidae, a group of Coleoptera with unique reproductive strategies, exhibit special leaf rolling behavior as part of their oviposition process. This behavior involves the creation of tightly closed leaf rolls, serving as protective enclosures for the development of eggs and larvae. Despite the considerable energy expenditure associated with this behavior, it significantly enhances the offspring’s survival rate. Compared with other insects, the reproductive strategy of Attelabidae leans more towards the K-selection end of the r-K breeding continuum. This distinctive oviposition behavior is believed to be connected to a reduction in the parasitic rate of parasitic wasps, indicating diverse reproductive strategies among different weevil groups. These may include laying multiple eggs in a single leaf roll to ensure offspring survival [8,9] or employing the strategy of cutting off petioles, causing more leaf rolls to fall on the ground and thereby avoiding parasitic wasps [10].

The species *A. dimidiatus* of the Attelabidae family is widely distributed in China and commonly found on *Rosa multiflora*, *Rosa chinensis*, and other plants. The insect feeds on leaves and creates compact leaf rolls for spawning, with its offspring completing a series of developmental stages within these structures before emerging as mature insects. Research indicates that *A. dimidiatus* can evade parasitic wasp parasitism by shifting between different host plants at various stages of occurrence and varying the number of eggs laid. While previous studies have contributed to understanding *A. dimidiatus* through behavioral observation and morphological identification, there is a conspicuous absence of genome sequencing studies on Attelabidae. Consequently, the molecular mechanism behind the evolution of leaf rolling behavior remains uncertain. Therefore, this study aimed to address this gap by sequencing and assembling the entire genome of *A. dimidiatus*, to elucidate the molecular mechanism underpinning the evolution of its leaf rolling behavior. This investigation sought to provide genomic resources necessary for phylogenetic analysis and comparative genomics research of Attelabidae.

## 2. Materials and Methods

### 2.1. Sampling

Samples of *A. dimidiatus* were collected in June 2022 from Tianxiang Meigui Resort in Wenjiang District, Chengdu City, Sichuan Province (30.70° N, 103.78° E, 542 m above sea level) for this study. The collected samples were then loaded into 1.5 mL centrifuge tubes, quick-frozen with liquid nitrogen, and stored in an ultra-low-temperature refrigerator at −80 °C.

### 2.2. Library Construction and Sequencing

DNA was extracted from the frozen samples to construct Illumina libraries, subsequently sequenced with the Illumina HiSeq 4000 platform (Novogene, Tianjin, China). Additionally, Nanopore libraries were created and real-time single-molecule sequencing was performed using Nanopore GridION X5/PromethION (Oxford Nanopore Technologies, Oxford, UK). RNA was also extracted to construct RNA libraries, sequenced using the Illumina high-throughput sequencing platform NovaSeq 6000 (Illumina, Inc., San Diego, CA, USA) with a sequencing strategy of PE150. To further improve the quality of the *A. dimidiatus* genome, optimization of the routine steps of Hi-C library [11,12,13] construction was carried out. This involved cutting frozen samples into small pieces and crosslinking them in EP tubes containing formaldehyde. Subsequent steps included digestion using 10× NEBuffer and the restriction enzyme (Dpn II), followed by biotin labeling. The Hi-C library was then completed, involving detection, repair, Polymerase Chain Reaction (PCR) amplification, and purification. Paired-end sequencing was performed on the Illumina HiSeq X10 platform (Illumina Inc., San Diego, CA, USA) with a read length of 150 bp. The sequencing data underwent processing to remove low-quality reads using fastp [14] in order to obtain clean reads. These clean reads were then used for subsequent K-mer evaluation, genomic error correction, auxiliary assembly, and other analyses. Meanwhile, the raw sequencing data from the Nanopore platform was subjected to filtering and quality control to obtain pass reads with a standard mean_qscore > 7 for subsequent assembly.

### 2.3. Genomic Prediction

Flow cytometry was utilized to assess the size of the *A. dimidiatus* genome, and de novo assembly estimated genome sizes before formal assembly, typically through K-mer analysis [15]. The study employed JellyFish v2.3.0 software to analyze filtered second-generation clean reads, and GenomeScope [16] was utilized for evaluating genome features such as size, duplication, and heterozygosity in *A. dimidiatus*.

### 2.4. Genome Assembly and Quality Assessment

NextDenovo v2.5.0 (https://github.com/Nextomics/NextDenovo, accessed on 22 January 2023) was employed for error correction and assembly of third-generation sequencing data from Nanopore, and heterozygous contigs were removed using purge_dups v1.0.0. Subsequently, Nextpolish [17] was used to refine the preliminary assembly results through second-generation Illumina data, and two rounds of iteration were implemented to enhance assembly quality further. Hi-C technology was implemented for assisted assembly, with Juicer [18] using the BWA program for comparing Hi-C reads to the initial genome sketch sequence and filtering the sequence using restriction endonuclease (Dpn II) tags. Chromosome-level assembly results were achieved using 3D DNA [19], and the obtained assembly results were visualized using Juicebox (v1.11.08). Moreover, the study employed minimap2 [20] and SAMtools [21] for progress evaluation and utilized Benchmarking Universal Single-Copy Orthologs (BUSCO) [22] to assess genomic integrity.

### 2.5. Genome Annotation

The identification of long terminal repeat retrotransposons (LTR-RTs) and tandem repeats was carried out using the LTR_finder v1.05 and TRF software, respectively. Homology alignment was performed by searching repeat sequences in the Repbase database using RepeatMasker v4.0.5 [23] and RepeatProteinMask v4.0.94. Additionally, RepeatModeler v1.0.4 was used to construct a species-specific model, and de novo prediction of repeat sequences was carried out using Augustus v3.4.0 [24], which involved masking repetitive sequences in the assembled genome. Genomic fasta files and annotated gff files of *Sitophilus oryzae* Linnaeus, *Ips. Typographus* Linnaeus, *I. nitidus* Eggers, *Hypothenemus hampei* Ferrari, *Rhynchophorus ferrugineus* Olivler, and *Dendroctonus ponderosae* Hopkins were downloaded from the NCBI and InsectBase2.0 databases. Subsequently, six de novo prediction models were constructed using Augustus, and protein sequences of these insects, along with *Leptinotarsa decemlineata* Say and *Tribolium castaneum*, were aligned with the assembled genomes of *A. dimidiatus* using TblastN v2.12.0 [25], and the BLAST hits were merged using Solar software. GeneWise [26] was then employed to predict the complete gene structure based on the corresponding gene region of each BLAST hit. The transcriptome-assisted prediction involved aligning the transcriptome reads to the masked repeat sequences using Hisat2 [27], followed by sorting and conversion using SAMtools [21] and assembly using StringTie [28]. Subsequently, genes were predicted using TransDecoder [29]. The results were integrated by EvidenceModeler (EVM) [30] and optimized by PASA to obtain non-redundant prediction results of the *A. dimidiatus* genome. The quality of the prediction results was assessed based on gene integrity and structure characteristics. Predicted protein sequences were compared to various databases, including KEGG, SwissPort, TrEMBL, NMBL, and NR, using BLASTP v2.2.26 with an E-value < 1 × 10^−5^. Alignment of the protein sequence with the Interpro and Gene Ontology (GO) databases was performed using InterProScan [31]. Based on the above two software comparisons to the databases, relevant functional and metabolic pathway information was obtained.

### 2.6. Comparative Genomes

The study focused on eight species, including six Curculionoidea species (*Sitophilus oryzae* (GCA_002938485.2), *Ips typographus* (GCA_016097725.1) [32], *I. nitidus* (GCA_018691245.1), *Hypothenemus hampei* (GCA_013372445.1) [33], *Rhynchophorus ferrugineus* (GCA_014462685.1) [34], and *Dendroctonus ponderosae* (GCA_000355655.1) [35]), together with *Tribolium castaneum* (GCA_000002335.3) [36] and the outgroup *Drosophila melanogaster* (GCA_000001215.4) [1], which were selected as reference species as they were all the Curculionidae species with annotated genes that were known. Annotation results and reference species’ protein files were obtained from the NCBI and InsectBase2.0 databases. Subsequently, a cluster analysis of protein sequences was conducted using OrthoFinder [37], and the sequences alignment of all single-copy homologous genes of the eight species was performed using Muscle software, followed by applying Gblocks software (Castresana 2000) to eliminate unconserved and unreliable regions. Subsequently, the filtered single-copy homologous genes from each species were merged to create a concatenated sequence, which was then converted into a phylip format file. Based on the PROTGAMMAWAG model, a maximum likelihood tree was constructed using RAxML [38]. During the tree construction, the species exhibiting either the earliest differentiation or the most distant genetic relationship were typically employed as the outgroup. In this study, *Drosophila melanogaster* was chosen as the outgroup. By running 1000 iterations on various heuristic trees, the best maximum likelihood tree and bootstrap evaluation values for each node were determined. The divergence times of various species were estimated using the MCMCTree program in PAML (Phylogenetic Analysis by 35 Maximum Likelihood) [39], based on the evolutionary tree topology generated by RAxML software. This involved analyzing the degree of differentiation between sequences and applying the molecular clock to estimate the divergence time of each node accurately. To enhance the reliability of these findings, fossil data from the TimeTree database (http://www.timetree.org/, accessed on 22 January 2023) were utilized as calibration points. MCMCTREE in PAML’s program is employed to estimate the origination and divergence times of nodes on the phylogenetic tree using Bayesian statistical methods and correct the fossil record data. After correcting the fossil time of the existing data, the posterior mean, 95% confidence interval, and the 95% confidence interval of the highest posterior density converged. Examples include the estimated divergence time of 3.58 million years for the beetle, and a range of 68.6–85.8 million years for the rice weevil and red palm weevil. Additionally, the divergence time between Coleoptera and Diptera was estimated to be within the range of 3.267–3.4 million years. Additionally, CAFÉ software was employed to establish the relationship matrix of the size of each gene family in different species. The expansion and contraction of the *A. dimidiatus* gene family were identified using the phylogenetic tree constructed by the single-copy homologous gene set, along with the estimated species divergence time. Furthermore, gene family selection pressure analysis was conducted using all single-copy orthologous genes identified in the eight species. Positively selected genes and genes undergoing rapid evolution were pinpointed using PAML. Following this, the amino acid sequences of expanded and contracted genes, positively selected genes, and genes undergoing rapid evolution in the *A. dimidiatus* gene family were extracted using self-written scripts. GO function and KEGG pathway annotations were carried out using the eggNOG-mapper (http://eggnog-mapper.embl.de/, accessed on 22 January 2023), and the annotation results were compiled using TBtools [40] to obtain the final result file. Finally, the clusterProfiler package in R-studio [41] was utilized for enrichment analysis and visualization of the results.

## 3. Results

### 3.1. Sequencing Data Statistics

Using the Illumina platform, a total of 163,325,898 clean reads were obtained, amounting to 49 Gb of valid data. Hi-C yielded 240,226,000 clean reads, comprising 72.07 Gb of valid data, while transcriptome sequencing filtering generated 78,651,024 clean reads, totaling 11.8 Gb of valid data. Subsequently, quality control measures were applied, assessing base quality and identifying that the Q20 ratio of the three sequencing results exceeded 97%, and the Q30 ratio reached 92%. Following quality filtering, 3,893,843 pass reads were recorded, accounting for approximately 49.34 Gb of valid data, with an average read length exceeding 10 Kb. The read length N50 was found to be 23.34 Kb, with the longest read reaching 179 Kb. Additionally, flow cytometry analysis indicated an average genome size of 581.993 ± 8.712 Mb for the three samples. Subsequent analysis of the *A. dimidiatus* genome revealed a predicted genome size of 720,818,244 bp, with a genome heterozygosity of 2.16%. The length of the repetitive sequences was estimated to be 518,659,763 bp, constituting approximately 71.95% of the genome. Moreover, the final genome size at the chromosome level was determined to be 619,259,538 bp, with 587.90 Mb (approx. 95%) of the sequence successfully mapped onto 14 chromosomes. Notably, the genome encompasses 1425 scaffolds, exhibiting a GC content of 33.89%, with a maximum length of 123,830,744 bp, an average of 434,568 bp, and an N50 of 50.89 Mb. Finally, the genome integrity was assessed using BUSCO at the chromosome level, with the semi-black elephant genome achieving an integrity score of 95.1%.

### 3.2. Genome Annotation

**Figure 1:**
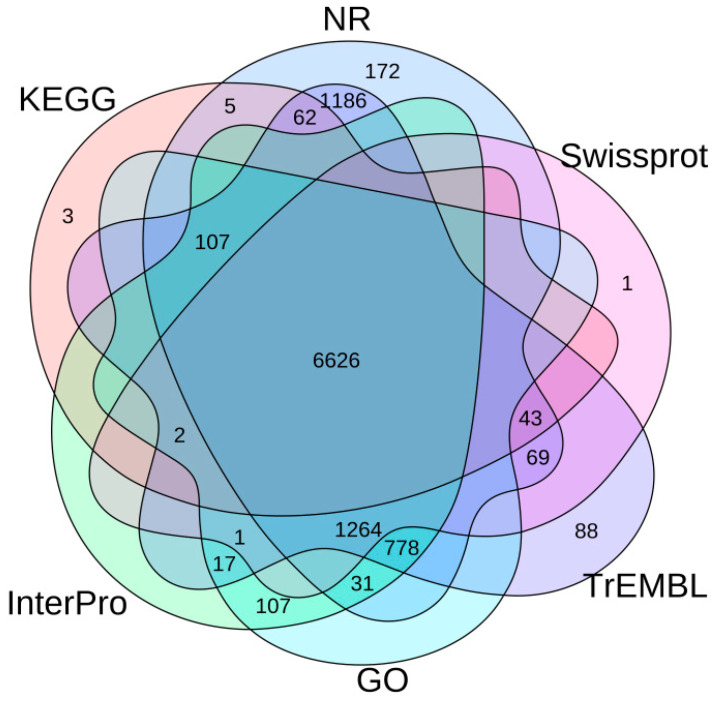
The statistics of functional annotation of the *A. dimidiatus* protein-coding genes based on different databases.

The annotation results for genome repeats indicated that *A. dimidiatus* displays a high proportion of repeats, accounting for 401,145,550 bp (64.78% of the genome size), with transposable elements being the most abundant at 63.61%. The analysis also revealed 12,572 predicted genes with an average gene length of 25,336.1 bp and an average exon length of 239.76 bp, achieving a gene prediction completeness of 94.2% (Table 1). Subsequently, the predicted genes were cross-referenced with six databases to procure functional and pathway annotation information (Figure 1), resulting in 82.59% of the predicted genes being validated by protein function databases.

### 3.3. Comparative Genomes

**Figure 2:**
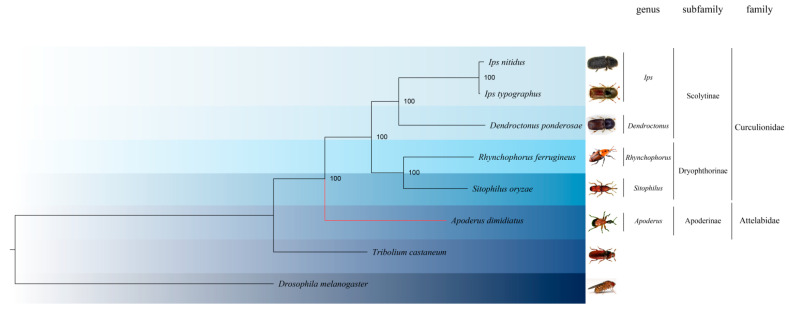
The phylogenetic tree of beetles Curculionidae and Attelabidae based on single-copy-homologous genes. Values in nodes indicate confidence interval. Red shows the position of *A. dimidiatus*.

The gene family clustering analysis by OrthoFinder for the eight species of insects involved a total of 153,762 genes, with approximately 84.3% (129,598 genes) being organized into 19,036 gene families, representing an average of 6.8 genes per family. Notably, 3744 of these gene families were found to be common to all species, with 1690 of them being single-copy gene families. *A. dimidiatus*, specifically, was found to have 11,597 genes organized into 8299 gene families, and of these, 1536 genes in 292 gene families were unique to *A. dimidiatus*. Utilizing the PROTGAMMAWAG model, a phylogenetic tree was constructed using RAxML software for the single-copy homologous genes of the eight species (Figure 2). 

**Figure 3:**
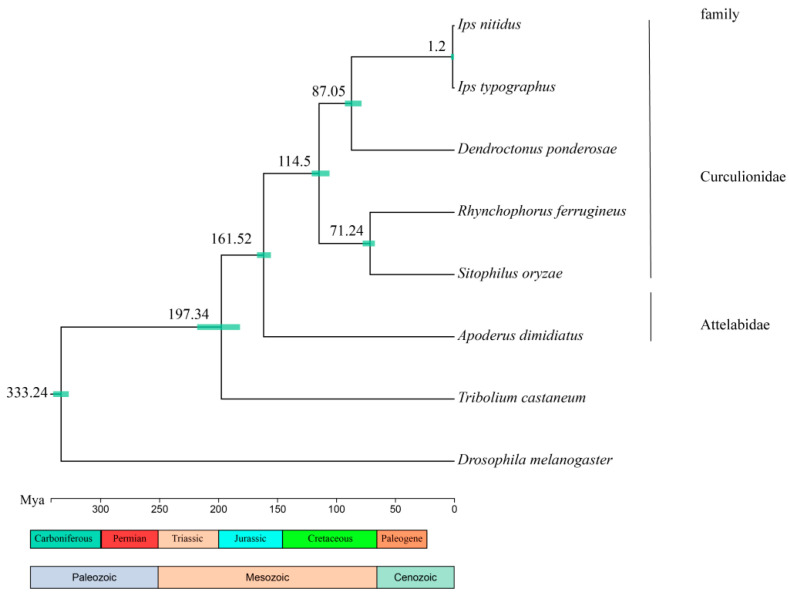
Divergence time tree of *A. dimidiatus* and other beetle species. The values in the nodes represent divergence time, and the bars represent era.

**Figure 4:**
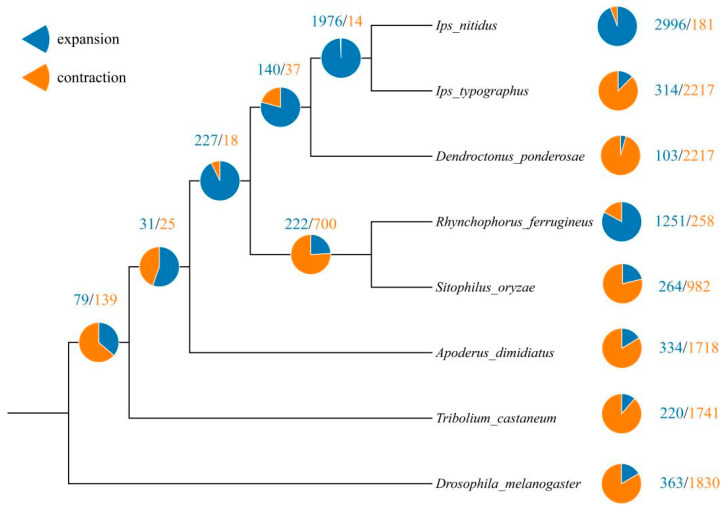
The results of family numbers of gene expansions or contractions based on *A. dimidiatus* and seven other species.

The longer branch lengths in the evolutionary tree indicate a faster evolutionary rate, which is observed in the genome of *A. dimidiatus*. This convergence of results suggests that the calculated data tends to be saturated and effective. Notably, the evolutionary analysis indicates that Diptera diverged from Coleoptera approximately 333.24 million years ago, Curculionoidea diverged from Tenebrionoidea around 197.34 million years ago, and Attelabidae diverged from Curculionidae around 161.52 million years ago (Table 2 and Figure 3). Furthermore, the genome of *A. dimidiatus* contained 334 expanded genes and 1718 contracted genes. Additionally, 31 gene families in the ancestral node of Curculionoidea underwent expansion, while 25 gene families underwent contraction (Figure 4).

**Figure 5:**
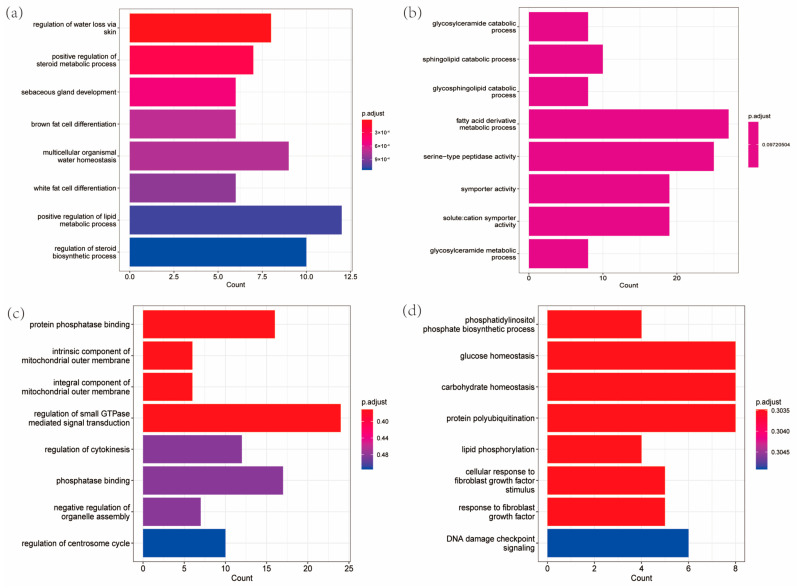
The gene families in *A. dimidiatus*. (**a**) The GO enrichment of expanded gene families. (**b**) The GO enrichment of contracted gene families. (**c**) The GO enrichment of positively selected genes. (**d**) The GO enrichment of papidly evolved genes.

The expanded gene families in *A. dimidiatus* were mainly linked to the functions of skin water loss regulation, multicellular in vivo water homeostasis, and steroid synthesis and metabolism, as indicated by the results of the GO enrichment analysis (Figure 5a). Conversely, the contracted gene families were mainly associated with the functions of glycosyl ceramide catabolism, sphingolipid catabolism, fatty acid derivative metabolism, and serine protease activity (Figure 5b). Moreover, the family of genes that underwent expansion in the ancestral nodes of Curculionoidea was found to be mainly associated with functions such as juvenile hormone esterase activity and regulation of synaptic activity. On the other hand, the family of genes that underwent contraction was primarily associated with functions such as alcohol esterification, development of tarsal glands, palmitoyl CoA9 desaturase activity, and monounsaturated fatty acid synthesis and metabolism. After identifying 1690 single-copy direct homologous genes, 540 positively selected genes and 106 rapidly evolving genes with significant signals were screened following data filtering at FDR < 0.05. The GO enrichment analysis of the positively selected genes in *A. dimidiatus* revealed enrichment in functions such as phosphatase binding, mitochondrial inner membrane composition, mitochondrial outer membrane composition, GTPase-mediated signaling, cell division, and organelle assembly (Figure 5c). Conversely, the GO enrichment of the rapidly evolving genes was associated with functions such as glucose homeostasis, carbohydrate in vivo equilibrium, protein ubiquitination, and DNA damage signaling (Figure 5d). Additionally, there were 21 genes belonging to rapid evolution in the ancestral branch node of Curculionoidea, and 781 genes were under positive selection. They could be enriched into the pathway by GO enrichment. Notably, rapidly evolving genes were mainly associated with synapses, while genes subject to positive selection were predominantly linked to ATP binding.

## 4. Discussion

K-mer analysis revealed that the *A. dimidiatus* genome harbors a significant number of repeat sequences and exhibits a high heterozygosity rate. The presence of numerous repetitive sequences and high heterozygosity are crucial determinants of insect genome assembly quality. The impact of heterozygosity can be mitigated by exploiting haploid males (Hymenoptera) [42] for sequencing or through controlled breeding [43]. However, the viability of these approaches is constrained by the challenge of maintaining *A. dimidiatus* in laboratory settings, as the sequencing samples were sourced from the wild. Consequently, the high heterozygosity observed in *A. dimidiatus* genomes is attributed to the infeasibility of implementing the abovementioned methods.

Only one study has been conducted on the reference genome of Attelabidae, which succeeded in assembling a reference genome of 428 Mb for the hazel leaf-roller weevil (*Apoderus coryli)* [7]. In contrast, our study assembled a reference genome of 619.26 Mb for *A. dimidiatus*, which was found to be 190 Mb larger than that of *A. coryli*, suggesting species-specific variations in genome size within the family. Interestingly, our findings revealed that the assembled *A. dimidiatus* genome size was smaller than that predicted by K-mer analysis. This discrepancy could be attributed to the K-mer analysis method being similar to the assembly principle with its simple sequence-based splicing strategy [44]; the high heterozygosity of the *A. dimidiatus* genome, leading to redundancy during splicing, exceeded the predicted size. Furthermore, compared with the genome size predicted by flow cytometry, our assembly result slightly exceeded the expected size, suggesting that the redundancy of sequences during the assembly process inflated the assembly size [45]. The presence of redundant sequences in the initial assembly was noted; however, after de-redundancy, error correction, and Hi-C-assisted assembly, the percentage of multi-copy genes in the reference genome of *A. dimidiatus*, as assessed by BUSCO, was found to be low (1.5%), indicating that the influence of redundant sequences on the final results was effectively eliminated through the more comprehensive assembly process in our study. Additionally, it is noteworthy that *Drosophila melanogaster*, which was used to predict the genome size via flow cytometry [46], was not considered in our study, suggesting a potential slight bias in the assessed size. Notably, the assembled sequences were successfully mapped onto 14 hypothetical chromosomes using Hi-C technology, consistent with previous studies that revealed the presence of 14 pairs of chromosomes (2*n* = 28) [47,48] in species of the genus *Apoderus*. This further supports the assertion that the chromosome number remains consistent mainly within the same genus [49,50,51]. The high quality of the chromosome-level genome assembly of *A. dimidiatus* was demonstrated through a comprehensive quality assessment, indicating improvements in terms of continuity, consistency, and completeness. This study provides a robust foundation for subsequent gene annotation and genetic analysis, offering an essential reference for the genome assembly of Attelabidae and closely related species. In conclusion, our study has successfully obtained a high-quality reference genome for *A. dimidiatus*, contributing to the advancement of genetic research in this species and related taxa.

The differences in genome size among different insect species are attributed to variations in the number of repetitive sequences resulting from amplifications, deletions, and differentiation [52]. In this study, a discrepancy of approximately 190 Mb in size between the genomes of *A. dimidiatus* and the hazel leaf-roller was observed in the assembly results. Furthermore, the genome annotation process not only involved annotating the assembled *A. dimidiatus* genome with repetitive sequences but also entailed downloading a fasta file of the hazel leaf-roller genome from the NCBI database and annotating the repetitive sequences of this genome. Upon comparing the repeat sequence information of the two species, it was revealed that the predicted length of repeat sequences in the *A. dimidiatus* genome was 401.15 Mb, while that in the hazel leaf-roller was 261.55 Mb, resulting in a disparity of around 140 Mb between the two species, primarily due to transposable elements. This finding aligns with previous studies that have indicated the accumulation of transposable elements as a contributory factor to changes in genome size [53]. Additionally, a high abundance of transposons was predicted in *A. dimidiatus.*

The study established precise developmental relationships among species by constructing developmental trees based on the direct lineage of single-copy homologous genes. The results revealed that the genetic relationships within the family align with previous research findings. Using fossil time correction, the study determined the divergence time between species. Specifically, the divergence time between Attelabidae and Curculionidae was estimated to be 161.52 million years ago (Mya), which is in line with previously reported estimates of 156 Mya [54] and 162.2 Mya [55]. In addition, the GO enrichment analysis of the expanded gene family of *A. dimidiatus* identified their involvement in regulating skin water loss, sebaceous gland development, water balance in multicellular organisms, adipocyte differentiation, lipid metabolism, and steroid synthesis and metabolism. The expansion of the gene families in *A. dimidiatus* can be attributed to the species’ specific ecological adaptations and life history traits. For instance, the female adult cuts off material flow between the leaves and the host plant. The entire developmental process of the offspring, from egg to adult, occurs within the leaf roll. As time progresses, the water content in the leaves decreases, imposing limitations on larval water intake. Consequently, *A. dimidiatus* larvae maintain water balance by secreting lipids to reduce epidermal water evaporation and regulate the extracellular fluid’s osmotic pressure. Furthermore, the leaf rolls, which serve as a shelter for the offspring, have limited nutrients, prompting them to expedite their development to locate new food sources as adults. The offspring develop into adults within approximately three weeks, largely facilitated by the secretion of a substantial amount of ecdysteroids by the larvae to induce molting, and the differentiation of adipocytes to form adipose cells, which constitute the fat bodies storing energy to sustain consumption during the pupal stage, ultimately culminating in their successful fledging. 

The contracted gene families of *A. dimidiatus* were subjected to GO enrichment analysis, which revealed their involvement in several essential functions. These gene families are primarily associated with serine protease activity, solute transmembrane transport, fatty acid derivative metabolism, sphingolipid catabolism, ceramide catabolism, and other functions. Serine protease activity is crucial in organisms’ physiological and pathological processes, as it participates in enzyme cascade reactions [56]. Notably, in insects, serine proteases also contribute to innate immunity. However, *A. dimidiatus* offspring, when faced with parasitism by parasitoids, do not survive as effectively as other insects, possibly due to the contraction of the gene family regulating serine protease activity. This contraction may result in an inadequate immune response, rendering the offspring susceptible to parasitism. Additionally, fatty acid derivatives, released as plant volatiles in response to leaf damage or insect bites, enhance plant resistance to insects [57]. *A. dimidiatus* exhibits a reduction in the genes related to fatty acid derivative metabolism, possibly because female adults disrupt material exchange between the leaf rolls and the host plant during leaf rolling. Consequently, larvae feeding on the rolls do not stimulate the host plant to secrete secondary metabolites such as fatty acid derivatives, leading to the contraction of the relevant gene family. Sphingolipid metabolism is closely linked to organism survival and reproduction. Ceramide is located at the center of sphingolipid metabolism [58]; studies have shown that abnormal accumulation of ceramide can negatively impact insect reproduction, particularly affecting female adults of *N. lugens*. This abnormal accumulation results in delayed maturation of oocytes, decreased oviposition, and diminished offspring survival rate [59]. Insects exhibit characteristics of being small, fast-growing, and short-lived, leading them to adopt the “r-selection” reproductive strategy emphasizing high fecundity to ensure population continuation and success through quantity rather than quality. On the other hand, the “k-selection” strategy focuses on producing a smaller number of offspring but with better mechanisms to protect them, thereby emphasizing quality over quantity. The analysis of this study reveals that the gene family related to ceramide catabolism function has reduced, potentially leading to ceramide abnormalities in the ovary, consequently affecting the reproduction of female adults and reducing egg production. To safeguard population continuation, the semi-black curly elephant developed the behavior of curling leaves to hide its offspring from environmental changes and natural enemies, ensuring their protection. This behavior has evolved over time, culminating in a sophisticated and distinctive reproductive behavior of leaf roll formation.

The regulatory center of cellular life activities is the reversible phosphorylation of proteins, a process primarily mediated by protein phosphatases and protein kinases. Protein phosphatases catalyze the dephosphorylation of phosphorylated proteins, resulting in the production of free phosphate molecules [60]. Reversible phosphorylation not only regulates protein activity through post-translational modification of proteins, but also plays a crucial role in the conversion of ATP to ADP [61]. Our study revealed that the positively selected genes of *A. dimidiatus* are predominantly associated with phosphatase binding, inner mitochondrial membrane composition, outer mitochondrial membrane composition, GTPase-mediated signal transduction, cell division, organelle assembly, and other functions. These genes may significantly contribute to biological mitochondrial synthesis and ATP production. The labor-intensive leaf rolling behavior of *A. dimidiatus* female adults demands substantial energy, potentially driving the synthesis of more mitochondria and subsequent ATP production to fulfill the organism’s energy requirements.

## 5. Conclusions

The study assembled a high-quality *A. dimidiatus* genome using Illumina and Nanopore sequencing platforms. A hypothesis for the expanded gene family of *A. dimidiatus* is their function association with the regulation of skin water loss, sebaceous gland development, water balance in multicellular organisms, adipocyte differentiation, lipid metabolism, steroid synthesis, and metabolism, which are deemed crucial for the survival of *A. dimidiatus* in leaf rolls. On the other hand, the contracted gene families are mainly related to serine protease activity, solute transmembrane transport, fatty acid derivative metabolism, sphingolipid catabolism, and ceramide catabolism. This could imply that the contraction of these gene families contributes to the decline in egg production and the low survival rate of offspring, forcing the female adults to develop the unique behavior of leaf rolling. Moreover, the study identified 106 rapidly evolved genes, primarily associated with glucose homeostasis, carbohydrate homeostasis, protein ubiquitination, and DNA damage signals. Furthermore, 540 genes were significantly under positive selection pressures in evolution, mainly related to phosphatase binding, inner mitochondrial membrane composition, outer mitochondrial membrane composition, GTPase-mediated signal transduction, cell division, and organelle assembly. It can be hypothesized that these positively selected genes play a crucial role in the energy supply of *A. dimidiatus*.

## Figures and Tables

**Table 1:** Statistics of the *A. dimidiatus* gene prediction.

Type	Parameter
Gene number	12,572
Average gene length (bp)	25,336.1
Average CDS length (bp)	1495.71
Average exon number	6.24
Average exon length (bp)	239.76
Average intron length (bp)	4551.09
BUSCO (%)	94.2

**Table 2:** The inspection data of MCMCTREE for the divergence time.

Node/Status	Value
t_n9	3.3327 (3.2670, 3.3998) (3.2672, 3.4000) 0.1328 (Jnode 14)
t_n10	1.9735 (1.8179, 2.1827) (1.8151, 2.1785) 0.3635 (Jnode 13)
t_n11	1.6151 (1.5519, 1.6707) (1.5531, 1.6717) 0.1187 (Jnode 12)
t_n12	1.1451 (1.0454, 1.2032) (1.0557, 1.2079) 0.1522 (Jnode 11)
t_n13	0.8704 (0.7786, 0.9234) (0.7845, 0.9264) 0.1419 (Jnode 10)
t_n14	0.0119 (0.0018, 0.0318) (0.0004, 0.0273) 0.0268 (Jnode 9)
t_n15	0.7125 (0.6778, 0.7894) (0.6714, 0.7755) 0.1041 (Jnode 8)
mu	0.3136 (0.1621, 0.5640) (0.1394, 0.5156) 0.3761
sigma2	0.1612 (0.0439, 0.3923) (0.0250, 0.3407) 0.3157
lnL	−8.7116 (−14.3650, −4.6540) (−13.7190, −4.2850) 9.4340

## Data Availability

The raw sequence data reported in this paper have been deposited in the Genome Sequence Archive (Genomics, Proteomics & Bioinformatics 2021) in National Genomics Data Center (Nucleic Acids Res 2022), China National Center for Bioinformation/Beijing Institute of Genomics, Chinese Academy of Sciences (GSA: CRA016725) that are publicly accessible at https://ngdc.cncb.ac.cn/gsa, accessed on 22 January 2023.
